# Antioxidant Properties of Crude Extract, Partition Extract, and Fermented Medium of* Dendrobium sabin* Flower

**DOI:** 10.1155/2017/2907219

**Published:** 2017-07-06

**Authors:** Farahziela Abu, Che Norma Mat Taib, Mohamad Aris Mohd Moklas, Sobri Mohd Akhir

**Affiliations:** ^1^Department of Human Anatomy, Faculty of Medicine and Health Sciences, Universiti Putra Malaysia, 43400 Serdang, Selangor (Darul Ehsan), Malaysia; ^2^Bioprocessing and Biomanufacturing Research Centre, Faculty of Biotechnology and Biomolecular Sciences, Universiti Putra Malaysia, 43400 Serdang, Selangor (Darul Ehsan), Malaysia

## Abstract

Antioxidant properties of crude extract, partition extract, and fermented medium from* Dendrobium sabin *(DS) flower were investigated. The oven-dried DS flower was extracted using 100% methanol (w/v), 100% ethanol (w/v), and 100% water (w/v). The 100% methanolic crude extract showed the highest total phenolic content (40.33 ± mg GAE/g extract) and the best antioxidant properties as shown by DPPH, ABTS, and FRAP assays. A correlation relationship between antioxidant activity and total phenolic content showed that phenolic compounds were the dominant antioxidant components in this flower extract. The microbial fermentation on DS flower medium showed a potential in increasing the phenolic content and DPPH scavenging activity. The TPC of final fermented medium showed approximately 18% increment, while the DPPH of fermented medium increased significantly to approximately 80% at the end of the fermentation.* Dendrobium sabin *(DS) flower showed very good potential properties of antioxidant in crude extract and partition extract as well as better antioxidant activity in the flower fermented medium.

## 1. Introduction

*Dendrobium sabin *(DS) is one of 1200 Orchid species from* Dendrobium* genome which has not been explored and there has been very limited study on DS previously. The identification number given by Herbarium Centre, Universiti Putra Malaysia (UPM), Malaysia, for* Dendrobium sabin* flower was SK 1966/11. The elegant dark purple color of DS flower is expected to contain numerous phenolic compounds and high antioxidant capacities. The major active constituent in few* Dendrobium* genera which has been isolated previously by using high performance liquid chromatography-diode array detection (HPLC-DAD) method was phenols that consist of bibenzyl, phenanthrene, and fluorenone [1]. Fluorenone was found to be unique to* Dendrobium s*pecies only as it was not found in other Orchid genera [2]. Previous study on ethanol extract of stems of* Dendrobium nobile *showed that the extract has a significant DPPH scavenging activity better than vitamin C and the research group successfully isolated the bibenzyl derivative from the ethanolic* Dendrobium nobile *stem extract [3]. In another study, other antioxidant constituents known as alkyl ferulates and quercetin have been isolated from methanol extract of* Dendrobium moniliforme* Sw. and* Dendrobium tosaense* Makino, respectively [4]. Lectin, enzymes (chalcone synthase, sucrose synthase, and cytokine oxidase), and polysaccharides are the bioactive compounds that have been identified from* Dendrobium* species [5] previously.

Normal cell metabolism continuously produces dangerous by-product substances known as free radicals. Free radicals are groups of atoms with unpaired electrons and can be formed when oxygen interacts with certain molecules. These organic molecules are highly reactive radicals and can start a chain reaction once they formed in the body just like the dominoes effect. Due to the unstable electron, the radicals tend to bond with other molecules to capture the electron in order to become stable, thus destroying healthy tissue and further continuing the damaging process. The antioxidant assays chosen for this study are 1,1-diphenyl-2-picrylhydrazyl (DPPH), 2,2-azino-bis-(3-ethylbenzothiazoline-6-sulfonic acid) (ABTS), and ferric ion reducing antioxidant power (FRAP) assays which were used to measure the relative antioxidant ability to scavenge the free radicals produced in the reagents. These three assays are based on electron transfer (ET) reaction principle, where the color change will act as an indicator to the capacity of antioxidant in reducing the radicals [6].

Emotional and physical stress, poor diets, and even pollution can all contribute to free radical formation in our system. Natural antioxidants produced within the cells are acting energetically to neutralize the harmful effect of free radicals. However, the additional antioxidants substances are still required by the body to fight the worst consequence of free radicals [7]. Antioxidants are crucially required to eliminate the free radicals that could cause an adverse effect towards DNA, proteins, lipids, and other biomolecules in the human body system. The amount of the endogenous antioxidant enzymes produced by our body might be inadequate. Most research put a great interest and attention on potential natural antioxidants which can be found in natural sources due to the undesirable effects of synthetic antioxidants towards the human endogenous enzymes [8].

Fermentation is a conventional method to preserve something using microbial cultures. It has been found that fermented soybean paste in Korea has better total phenolic contents (TPC) and comprises stronger antioxidant capacity as compared to unfermented soybean because the fermentation is believed to aid in producing more aglycone isoflavone and malonylglycoside isoflavone [9]. Lactic acid fermentation, on the other hand, was proven to significantly improve the DPPH radical scavenging activity of peanut flour [10]. The soybean or cowpea that has been fermented with lactic acid bacteria also showed better effect on DPPH activity [11, 12]. The increase of DPPH capacity after fermentation process indicates that fermentation possibly has a great potential in producing some metabolites with superior radical scavenging activity [10]. Another fermentation study on* Coffee arabica* pulp extracts showed that fermentation did increase the free phenolic compound from 36% to 42% and thus shows higher antioxidant capacity [13]. The coffee pulp contains natural antioxidants like hydroxycinnamic acids which could be released during fermentation or enzymatic process. Fermentation also leads to microbial hydrolysis reaction and thus could increase the phenolic compound [14]. The structural breakdown of plant cell walls could be triggered by fermentation process and hence generate the synthesis of various antioxidant compounds. The pH condition plays a role in fermentation, where the content and structure of phenolic compound could be easily affected by pH. For example, anthocyanin is stable in pH 1-2, while quercetin is most active at pH 5.5. Catechin on the other hand is unstable in alkaline solution but stable in acidic condition [14].

## 2. Materials and Methods

### 2.1. Materials

*Dendrobium sabin* flowers were purchased from Sungai Buloh Nursery in Selangor (Darul Ehsan), Malaysia. The plant was sent to botanist of Universiti Putra Malaysia (UPM), Malaysia, for species identification and the voucher specimen obtained was SK 1966/11. Analytical grade solvents were used for the extraction. The solvents used in this study (ethanol, methanol, ethyl acetate, hexane, and acetic acid) were purchased from Systerm. The chemicals used throughout this study such as 1,1-diphenyl-2-picrylhydrazyl (DPPH), 2,2-azino-bis-(3-ethylbenzothiazoline-6-sulfonic acid) (ABTS), ferric chloride solution, Folin-Ciocalteu reagent, potassium ferricyanide solution, potassium persulfate, sodium dodecyl sulfate (SDS), gallic acid powder, and Trolox powder were purchased from Sigma-Aldrich and Merck.

### 2.2. Antioxidant Properties of Crude and Partition Extract

*(i) Preparation of Methanolic, Ethanolic, and Water Crude Extract. Dendrobium sabin* (DS) flowers were washed thoroughly and oven-dried at 40°C for 5 days until the flower became fully dried. The dried flowers remained purple in color and were then ground to fine powder using a stainless steel blender. The powdered form of oven-dried DS flowers was subjected to solid-liquid extraction by using three different solvents which are 100% methanol, 100% ethanol, and water by using the same extraction procedures. Each 1 g of dried DS flower sample was extracted by using 10 ml of solvents (100% methanol, 100% ethanol, and water) with solid to solvent ratio of 1 : 10 (w/v). The mixture of powdered flower and methanol was continuously swirled at 150 rpm in a shaker incubator for 2 hours at 37°C before being filtered using Whatman filter paper number 1. The residue was then reextracted twice following the same procedure. The collected filtrates were subjected to rotary evaporator to remove the entire methanol and finally the crude extract was formed and then stored at −20°C until further analysis.

*(ii) Preparation of Ethyl Acetate, Hexane, and Water Partition Extract*. The dried DS flower sample was divided to two parts; each part was extracted by using 100% methanol (C 100) and another one was using acidified methanol (C A). Then, solvent-solvent partition was carried out by using three different solvents with different polarity index which were 100% hexane (0 polarities), 100% ethyl acetate (4.4 polarities), and water (9 polarities) on each crude extract from 100% methanol and acidified methanol.

*Partition: Water and Hexane*. The crude methanolic extract obtained from extraction by 100% methanol (C 100) and acidified methanol (C A) was collected after being dried under vacuum in rotary evaporator. Every 1 g of crude methanolic extract was dissolved in 20 ml of distilled water with solid to solvent ratio of 1 : 20 (w/v) and then 20 ml of 100% hexane was added in a first separatory funnel. Ratio of 20 : 20 (v/v) of water and hexane was used for separating the nonpolar compounds. The separatory funnel was shaken for 2 minutes before being allowed to settle down for 2 hours at room temperature. The lower phase which was the water layer was withdrawn from the funnel's stopcock, while the hexane layer on the upper part was poured out from the funnel's stopper. The procedure was repeated for 5 times and the total hexane layer was collected. The collected hexane layers (H 100 and H A) were filtered and concentrated under vacuum in rotary evaporator to give the hexane layer crude extract (H 100 and H A).

*Partition: Water and Ethyl Acetate*. Ethyl acetate was added to the water layer obtained after the partition with hexane with 1 : 1 ratio (v/v) in the separatory funnel. The funnel was shaken for 2 minutes and left at room temperature for 2 hours to settle down. The water layer settled at the bottom layer was withdrawn from the stopcock, while the ethyl acetate layer settled on the upper layer was collected from the funnel's stopper. The procedure was repeated for 5 times and total ethyl acetate layer was collected. The collected ethyl acetate layer was filtered and concentrated under vacuum in rotary evaporator to give the ethyl acetate layer (EA 100 and EA A) crude extract. The water layers (W 100 and W A) were freeze-dried and have been properly sealed with parafilm and stored at −20°C until further analysis.

*(iii) Total Phenolic Content (TPC)*. Total phenolic content (TPC) was determined by using Folin-Ciocalteu reagent based spectrophotometric assay [15] with slight modifications. 10 mg of each crude and partition extract was individually dissolved in 1 mL of corresponding extracting solvent to produce the stock of sample solution at 10,000 ppm. The lower concentrations of sample at 500 ppm and 1000 ppm were prepared by diluting the stock sample solution. 0.5 ml of sample was put in a test tube and mixed with 2.5 ml of freshly prepared 10% Folin-Ciocalteu reagent, followed by an addition of 2.0 ml of 7.5% sodium carbonate after 5 minutes. Then, the resulting mixture was incubated at 40°C for an hour and absorbance was measured at 765 nm after incubation. Gallic acid was used as calibrated standard and results were expressed as milligram gallic acid equivalent per gram of dry weight (mg GAE/g dry weight). The content of phenolics for each extract was determined in triplicate, results were averaged, and data was reported as mean ± SD.

*(iv) DPPH Assay*. The free radical scavenging activity of each crude and partition extract was measured by 1,1-diphenyl-2-picrylhydrazyl (DPPH) assay [16] with slight modifications. Fresh methanolic solution of DPPH (0.2 mM) was prepared and incubated in the dark for 2 hours prior to the analysis. Each crude extract as well as the Trolox powder as the standard was dissolved individually in their corresponding extracting solvent. Then, 0.05 ml of each of these test sample solutions at various concentrations was transferred to 96-well plates. DPPH methanolic solution (0.195 ml) was then added to each well by using multichannel pipette. The absorbance of resulting mixture was measured at 540 nm by using microplate reader after incubation period of an hour in the dark. DPPH radical scavenging activity was expressed as Trolox equivalent per gram dry weight (mg TE/g dry weight) and calculated using the following formula:

(1) DPPH  radical  scavenging  activity=Absorbanceblank−AbsorbancesampleAbsorbanceblank×100.

*(v) 2,2-Azino-Bis-(3-ethylbenzothiazoline-6-Sulfonic Acid) (ABTS) Assay*. 2,2-Azino-bis-(3-ethylbenzothiazoline-6-sulfonic acid) (ABTS) radical cation scavenging activity of each crude and partition extract was conducted according to method described by a previous study [17]. For this test, ABTS reagent solution was freshly prepared by mixing 7 mmol of ABTS solution treated with 2.45 mmol of potassium persulfate. ABTS powder and potassium persulfate powder were individually dissolved with water to the required concentration and then were mixed together in a bottle. After 16 hours of incubation of resulting mixture in the dark at ambient temperature, the resultant dark blue color of ABTS reagent solution was diluted with distilled water until the absorbance reading reached 0.700 ± 0.005 at 734 nm. The Trolox and each crude and partition extract were dissolved individually in their extracting solvent to the concentration of 500 *μ*g/ml. Each test sample solution (0.1 ml) was mixed with 0.9 ml of ABTS stock solution. The absorbance was measured at 734 nm after 2 minutes of mixture reaction. The ABTS radical cation scavenging activity was expressed in milligram Trolox equivalent per gram of dry weight (mg TE/g dry weight) and was calculated using the following formula:

(2) ABTS  radical  scavenging  activity=Absorbanceblank−AbsorbancesampleAbsorbanceblank×100.

*(vi) Ferric Reducing Antioxidant Power (FRAP) Assay*. Ferric reducing antioxidant power(FRAP) assay was conducted according to the FRAP assay method [18]. This assay involved a reduction from Fe^3+^ to Fe^2+^ by electron transfer reaction. Gallic acid that acts as the calibration standard was dissolved in methanol and diluted to form standard concentration of 100 *μ*g/ml to 3.125 *μ*g/ml. All the crude and partition extracts were dissolved in their extracting solvent to the concentration of 500 *μ*g/ml. 1 ml of each dissolved extract was mixed with 5 mL distilled water, 1.5 ml HCL (1 M), 1.5 ml potassium ferricyanide solution (1%, w/v), 0.5 ml of sodium dodecyl sulfate (SDS) solution, and lastly 0.5 ml of ferric chloride solution (0.2%, w/v). Each reaction tube was then vortexed and incubated at 50°C for 20 minutes. The absorbance of each mixture was measured at 750 nm using spectrophotometer and the FRAP was expressed as milligram gallic acid equivalent per gram of dry weight (mg GAE/g dry weight).

### 2.3. Fermentation Process to Increase Antioxidant Properties

*(i) Preparation of Bacteria*. The DS flower was washed and soaked in tap water for 30 minutes to remove dirt and rinsed twice with running tap water. The fresh flower was blended using mortar to make a fine paste. 22.4 g of nutrient agar powder was dissolved in 800 ml of distilled water and autoclave at 121°C for 20 minutes. The agar solution was solidified in Petri dish and stored at 37°C. By using aseptic techniques, few loops of the blended flower were streaked on the agar plate and put in the 37°C oven incubator to allow the bacteria to grow.

*(ii) Fermentation of DSF*. Five grams of DS fresh flower was mixed with 100 ml of 1% glacial acetic acid by using laboratory blender. The mixture was then filtered by using Whatman filter paper to get the filtrates. The filtrate which was purple in color was the flower juice or is called the medium. The medium together with the conical flask and cotton wool that will be used during the fermentation process need to be sterilized by using an autoclave at 121°C. After disinfection procedure, the cultured bacterium that has been isolated from the DS flower was inoculated into the medium. The conical flask containing the medium and the bacteria was subjected to shaking incubator for 84-hour fermentation at constant speed of 200 rpm and temperature of 37°C. The collection of fermented flower medium was done every six hours (*T* = time of 6-hour interval) during the 84-hour fermentation process. The collected medium was analyzed for the pH and optical density for determination of bacterial concentration. The collected medium was stored at −20°C for further analysis. The fermented medium has also been analyzed for antioxidant capacity to determine the antioxidant production by fermentation process.

*(iii) pH Analysis and OD Measurement*. The collected fermented medium products were analyzed for the pH by using pH meter right after each time of collection (*T* = 6-hour interval). Optical density (OD) was used to determine cell count. The spectrophotometer's wavelength was set to 610 nm and distilled water was used as the blank. The absorbance reading of each fermented medium collected was taken. The OD was calculated by using the following formula:

(3) $$\begin{array}{c} OD=absorbance\;\;reading-blank. \end{array}$$

*(iv) TPC of Fermented Medium*. The freeze collected fermented medium was thawed to room temperature before being subjected to centrifuge at 4000 rpm for 2 minutes. The supernatant was collected for determining the phenolic content and antioxidant capacity. Total phenolic content was determined by using Folin-Ciocalteu reagent based on spectrophotometric assay [15] with slight modifications. 0.5 ml of fermented medium collected at various time intervals was put in a test tube and mixed with 2.5 ml of freshly prepared 10% Folin-Ciocalteu reagent, followed by an addition of 2.0 ml of 7.5% sodium carbonate after 5 minutes. Then, the resulting mixture was incubated at 40°C for an hour and absorbance was measured at 765 nm after incubation. Gallic acid was used as calibrated standard and results were expressed as milligram gallic acid equivalent per gram of dry weight (mg GAE/g dry weight). The content of phenolics for each fermented medium was determined in triplicate, results were averaged, and data was reported as mean ± SD.

*(v) DPPH of Fermented Medium*. The free radical scavenging activity of collected fermented medium was measured by 1,1-diphenyl-2-picrylhydrazyl (DPPH) assay [16] with slight modifications. Fresh methanolic solution of DPPH (0.2 mM) was prepared and incubated for 2 hours prior to the analysis. 0.05 ml of collected fermented medium and Trolox solution were transferred to 96-well plates. DPPH methanolic solution (0.195 ml) was then added to each well by using multichannel pipette. The absorbance of resulting mixture was measured at 540 nm by using microplate reader after incubation period of an hour in the dark. DPPH radical scavenging activity was expressed as Trolox equivalent per gram dry weight (mg TE/g dry weight) and calculated using the following formula:

(4) DPPH  radical  scavenging  activity=Absorbanceblank−AbsorbancesampleAbsorbanceblank×100.

## 3. Analysis

The data were analyzed using SPSS 19.0 and Tukey's multiple comparison test was used. Significant differences among the treatments and dosages were determined by *p* value with *p* being less than 0.001 (*∗∗∗*), 0.01 (*∗∗*), or 0.05 (*∗*). Data was presented as mean ± standard deviation (SD).

## 4. Results

### 4.1. Yield Extract and TPC of Crude Extract

The extraction yield and total phenolic content of DSF methanolic crude extract (DSF MCE), DSF ethanolic crude extract (DSF ECE), and DSF water crude extract (DSF WCE) are presented in Table 1. The extraction using water (DSF WCE) could extract nearly 46%, whereas extraction using ethanol (DSF ECE) could only extract approximately 7% yield. The total phenolic content shows the contrast, where the DSF MCE has the highest total phenolic content followed by DSF ECE and DSF WCE.

### 4.2. Antioxidant Properties of Crude Extract

**Figure 1:**
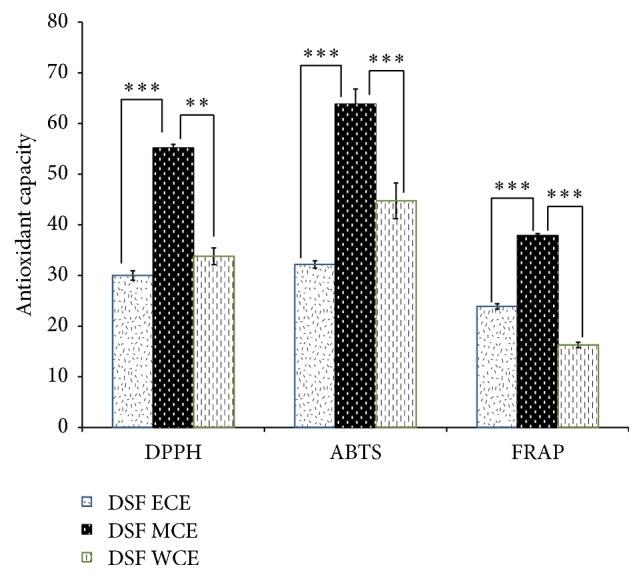
DPPH, ABTS, and FRAP scavenging activity of* Dendrobium sabin* flower's crude extract. The DPPH, ABTS, and FRAP results are presented as mean ± SD. Post hoc (Tukey's) test shows significant difference between DSF MCE and DSF ECE and DSF WCE at *p* < 0.001 (*∗∗∗*) in both FRAP and ABTS assays. However, in DPPH assay, DSF MCE shows significant different at *p* < 0.001  (*∗∗∗*) between DSF MCE and DSF ECE; meanwhile DSF MCE and DSF WCE are significantly different at *p* < 0.01 (*∗∗*).

The antioxidant assays (DPPH, ABTS, and FRAP) measure the relative antioxidant ability enclosed in DSF to scavenge the free radicals produced in the reagents.  Figure 1 presents the antioxidants capacity determined by DPPH, ABTS, and FRAP test methods for DSF MCE, DSF ECE, and DSF WCE. All crude extracts were reconstituted in their corresponding solvent prior to the experiment but all the blank, reagents, and controls (Trolox and gallic acid) were dissolved in methanol. DSF MCE possesses the highest antioxidant capacity as compared to DSF WCE and DSF ECE in all antioxidant assays (DPPH, ABTS, and FRAP). DSF WCE showed better antioxidant capacity than DSF ECE in DPPH and ABTS assays. However, in FRAP assays, DSF ECE performed better scavenging activity than DSF WCE. Statistical analysis shows significant difference between these 3 extracts on each of DPPH, ABTS, and FRAP assays.

### 4.3. Yield Extract and TPC of Partition Extract

Since DSF MCE showed the best performance in antioxidant capacity, it was further tested for partition extraction. 100% methanol was compared to acidified methanol in which both were used for extracting 40 g of grounded DSF, respectively.  Table 2 shows the percentage of extraction yield from 100% methanol (C 100) and acidified methanol (C A). The result shows that using acidified methanol could produce extra 3.5% yield which is better than using 100% methanol. Further solvent-solvent partition using hexane (H), ethyl acetate (EA), and water (W) on each C 100 and C A was carried out and thus produced different percentage of yield extract as shown in Table 2. Partition using water poses highest yields followed by ethyl acetate and the hexane. The TPC for partition extract are presented in Table 2. DSF crude extract which was extracted by 100% methanol contains more phenolic content as compared to crude extract from acidified methanol. Ethyl acetate could extract the highest TPC as compared to water and hexane. It can be concluded that extraction using 100% methanol followed by further solvent-solvent partition using ethyl acetate will produce the most phenolic compounds.

### 4.4. Antioxidant Properties of Partition Extract

**Figure 2:**
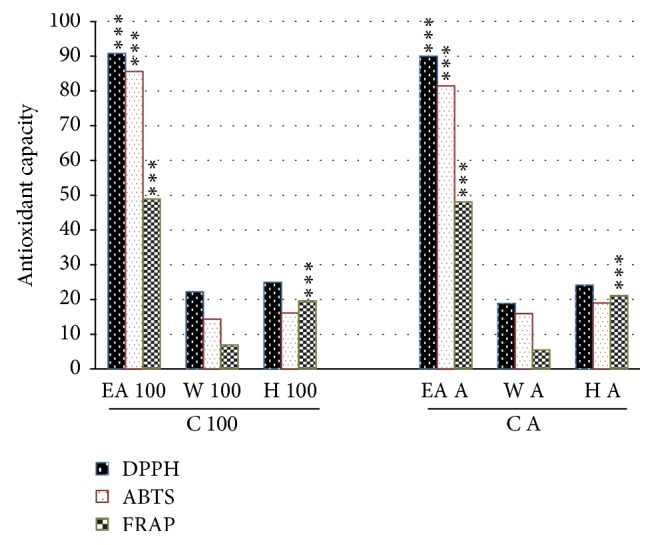
Antioxidant capacity assays (DPPH, ABTS, and FRAP) between partition extracts. The DPPH, ABTS, and FRAP results are presented as mean ± SD. Post hoc (Tukey's) test shows significant difference in all DPPH, ABTS, and FRAP assays between ethyl acetate extract (EA 100; EA A) with water layer extract (W 100; W A) and hexane extract (H 100; H A) at *p* < 0.001, respectively. In FRAP assay, water layer extracts (W 100 and W A) are also significantly different from hexane layer extracts (H 100 and H A) at *p* < 0.001, respectively. *∗∗∗* refers to *p* value < 0.001.

The antioxidant properties of partition extract are presented in Figure 2. Ethyl acetate partition extract has the highest antioxidant capacity as compared to water and hexane partition extract. It can be concluded that extraction using 100% methanol followed by further solvent-solvent partition using ethyl acetate will produce the best antioxidant extract.

### 4.5. Fermentation Process to Increase Antioxidant Properties

**Figure 3:**
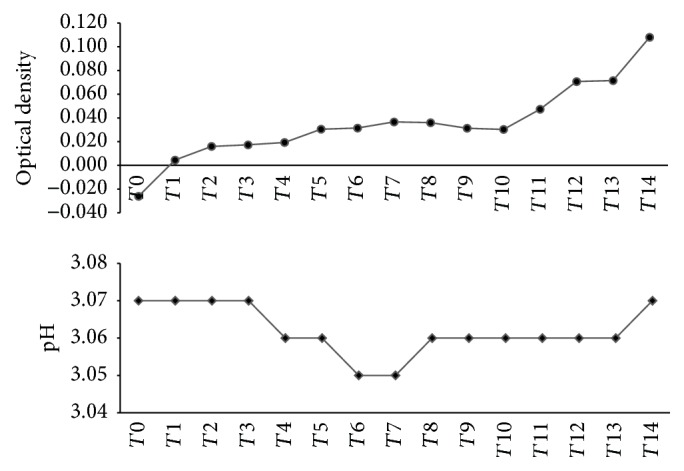
pH changes and optical density (OD) of the fermented medium over the 84-hour period. *T* is 6-hour interval time between collections of one sample to another.

There was some bacterial growth in the medium based on the pH changes of the collected medium and also the optical density result as presented in Figure 3, which showed an increasing trend of optical density (OD) at approximately 130%. The highest value of OD at *T*14 indicated that the medium contained the most bacteria in it after 84 hours of fermentation. *T*0 until *T*14 represented the 6-hour interval time to take out a volume of medium based on the duration of 84 hours of fermentation.

**Figure 4:**
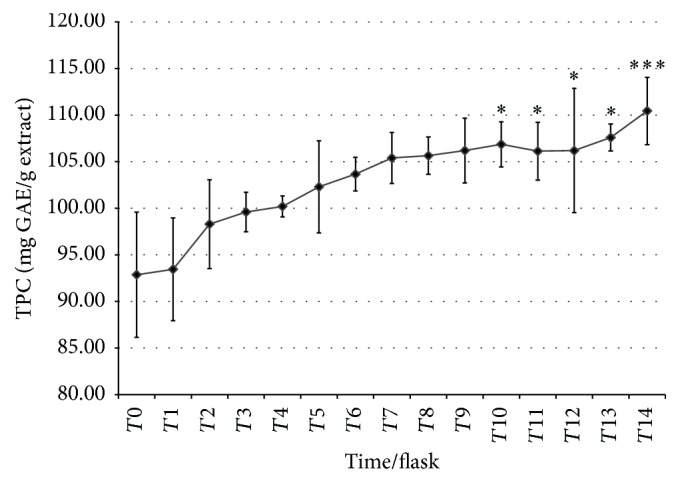
TPC of fermented medium from *T*0 to *T*14. The TPC results are presented as mean ± SD. Post hoc (Tukey's) test shows significant difference in TPC between *T*14 and *T*0 at *p* < 0.001 (*∗∗∗*); meanwhile *T*10, *T*11, *T*12, and *T*13 were significantly different from *T*0 at *p* < 0.05 (*∗*).

**Figure 5:**
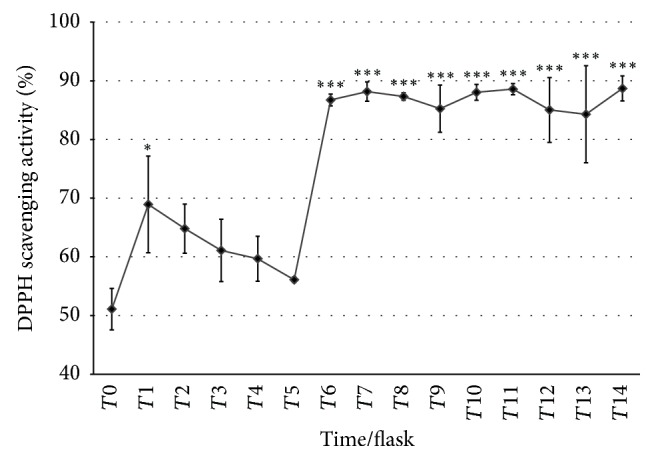
DPPH scavenging activity of fermented medium from *T*0 to *T*14. The DPPH results are presented as mean ± SD. Post hoc (Tukey's) test shows significant difference in DPPH between *T*6, *T*7, *T*8, *T*9, *T*10, *T*11, *T*12, *T*13, *T*14, and *T*0 at *p* < 0.001 (*∗∗∗*), respectively, while *T*1 was significantly different from *T*0 at *p* < 0.05  (*∗*).

Figures 4 and 5 showed the TPC and DPPH of the fermented medium which were collected every 6 hours within 84 hours of fermentation. The TPC was increased from time to time indicating that the fermentation of the* Dendrobium sabin* flower medium has positive effect in multiplying the phenolic content. Starting from *T*10, the TPC has a significant increase as compared to *T*0. The DPPH result on the other hand showed sudden increase at *T*1 but reduced until *T*5 before a sudden increase again at *T*6 and continued at the same rate until *T*14.

## 5. Discussions

### 5.1. Antioxidant Properties of Crude and Partition Extract

The extraction yield showed differences which suggest that different solvent used for extraction would extract different compounds depending on the polarity of the solvent, the chemical nature of extracted phenolic compound, the extraction method, plant matrix, and the presence of interfering substances [19]. Based on polarity scale, water has the highest polarity of 10.2 and methanol has polarity of 5.1, while ethanol has polarity of 4.3; hence, we can conclude that the more polar solvent will produce more extraction yield, where, in this study, DSF WCE has the highest yield extract, followed by DSF MCE and DSF ECE. Furthermore, protein and carbohydrates are more soluble in water than methanol and ethanol which could have contributed to the higher yield [20]. The extraction yield between 100% methanol (C 100) and acidified methanol (C A) also showed slight difference in yield percentage, where the C A produced extra 3.5% than C 100. Acidified methanol produced extra yield compared to 100% methanol alone due to the solvent combination of methanol and acid, where acid could destroy the cell membrane and thus dissolving and stabilizing more phenolic compounds especially anthocyanins [21]. The further solvent-solvent partition using hexane, ethyl acetate, and water on each C 100 and C A crude extract also gave different percentage on yield extract. Partition procedure using water posed highest yields followed by ethyl acetate and hexane. The difference was because water has the highest polarity capacity compared to hexane and ethyl acetate. The solvent polarity really plays a vital role in increasing phenol solubility [22].

The TPC of crude extract result shows that the DSF MCE has the significantly highest total phenolic content followed by DSF ECE and DSF WCE. The TPC result suggested that the polarity of the solvent has an effect on extracting different phenolic compounds from the DS flower and therefore would have different total phenolic content. Methanol extracted extra phenolic compound as compared to ethanol and water. Methanol and ethanol have similar polarity but gave distinctive result, where ethanol was less efficient than methanol. This was because ethanol has low solvation of antioxidant compound which was caused by the presence of longer ethyl radical than the methyl radical in methanol [19]. The phenolic compounds also have varieties in their chemical structure [23] and thus performed differently towards Folin-Ciocalteu method [24].

The TPC of partitioned crude extract showed that EA 100 and EA A both had very significantly high total phenolic contents as compared to water (W 100 and W A) and hexane (H 100 and H A). The solvent-solvent partition was done on methanolic crude extract using different polarity solvent from polar (water), semipolar (ethyl acetate), and nonpolar (hexane) solvents in order to acquire the faction of difference classes of phenolic compound. The water layer could extract polar compound (anthocyanins) with some flavonoids, while the ethyl acetate could extract phenolic compounds but not anthocyanin. A partition study done on* Dendrobium sonia *“Red Bom” showed that the water layer contained the highest phenolic compound as compared to ethyl acetate and hexane layer due to the presence of anthocyanins in the water layer [25]. This study showed the contrast result, where the ethyl acetate layer showed the highest total phenolic compounds compared to water and hexane. This contradictory result might be caused by the reduced amount of anthocyanins in water layer (W 100 and W A) due to procedure of drying the flower in the oven before the extraction process. It has been reported previously that the method of drying in the oven could reduce the anthocyanins at 35% to 39% on Carménère variety and Cabernet Sauvignon variety, respectively [26]. Thus, it can be concluded that the EA 100 and E A contain the highest phenolic content due to the ethyl acetate capability of extracting the phenolic compound from DS flower, while anthocyanin was reduced in water layer due to the procedure of drying in the oven.

**Figure 6:**
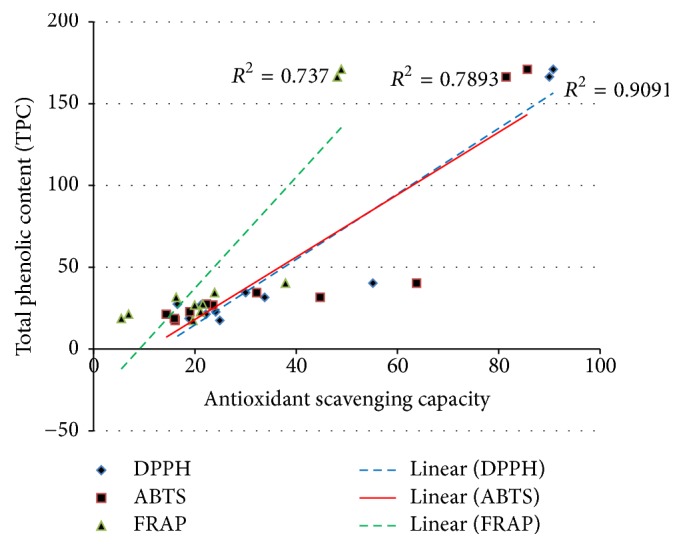
Correlation between TPC and antioxidant capacity (DPPH, FRAP, and ABTS).

The antioxidant assays (DPPH, ABTS, and FRAP) measured the relative antioxidant ability contained in DS flower to scavenge the free radicals produced in the reagents. All crude extracts were reconstituted in their corresponding solvent prior to the experiment but all the blank, reagents, and controls (Trolox and gallic acid) were dissolved in methanol. TPC and all antioxidant assays (DPPH, ABTS, and FRAP) showed a strong correlation between total phenolic content and the antioxidant capacity as shown in Figure 6, where the phenolic content acts as the major contributor for antioxidant capacity. The correlation between TPC and antioxidant capacity described by *R*^2^ value (correlation coefficient) of all crude extract and partition extract showed that TPC and DPPH have the highest correlation (*R*^2^ = 0.909), followed by TPC with ABTS and FRAP, respectively. TPC by Folin-Ciocalteu's reagent, DPPH, and FRAP involved the same electron transfer method during reaction which explains the good correlation between those three assays (TPC, DPPH, and FRAP) [3].

### 5.2. Fermentation Process to Increase Antioxidant Properties

It can be concluded that there is some bacteria growth in the medium based on the optical density result which shows an increasing trend. The highest value of OD at *T*14 indicated that the medium contained the most bacteria in it after 84 hours. The pH was also reduced during *T*4 until *T*13 which indicated that the fermentation process was commenced in the DS flower medium. Lactic fermentation on the Malaysian herbal tea was also found to reduce in pH during fermentation [27]. The pH could affect the phytochemicals during fermentation such as anthocyanin which is only stable at pH 1-2 [14].

The TPC got increased which indicated that the fermentation of the DS flower medium has positive effect in multiplying the phenolic content, where the TPC has a significant increase starting at *T*10. A study done on lactic fermented herbal tea was found to be high in TPC and antioxidant properties as compared to freshly prepared herbal tea, where FRAP and bleaching assay were used. The microbial fermentation process on tea exhibits a unique phytochemical profile compared to the unfermented tea because of the enzymatic reaction with substrates that produce more phenolic compound as the final product [27]. Fermentation also has been found to increase the free phenolic compound for about 6% in fermented coffee pulp because the pulp contains natural antioxidants like hydroxycinnamic acids which can be produced by fermentation or enzymatic process [13]. Both fermented herbal tea and coffee pulp showed higher antioxidant capacity too.

The DPPH result showed sudden increase at *T*1 but reduced until *T*5 before a sudden increase again at *T*6 and continued at the same rate until *T*14. The same trend for DPPH scavenging activity has been shown by a study of DPPH activity in fermented milk (yogurt), where the study found the drastic increase of the fermented milk as compared to unfermented milk followed by a linear trend with small deviation [28]. The fermentation causes few molecular changes in the milk which produce peptides, free amino and fatty acids, enzymes, and other compounds which have potential in redox balancing and thus increase the antioxidant capacity [28]. Fermentation induced the structural breakdown of plant cell walls with the aid of microbial hydrolysis reaction, releasing various antioxidant compounds [14]. Lactic acid fermentation on berries had also increased the total phenols, flavonoids, and anthocyanins at 5–10 times higher than nonfermented berries, while the DPPH scavenging activity had increased by 30% in fermented berries compared to nonfermented berries. The HPLC analysis on the fermented berries found increase in gallic acid, myricetin, and quercetin [29].

## 6. Conclusion

In summary,* Dendrobium sabin *(DS) flower demonstrated a very good potential property of antioxidant. The extraction of oven-dried DS flower using 100% methanol has shown that methanol could extract more phenolic content as compared to ethanol and water extraction, and thus 100% methanolic crude extract of DS flower (DSF MCE) has demonstrated the best antioxidant properties too. Further solvent-solvent partition on DSF MCE using ethyl acetate, water, and hexane showed ethyl acetate layer to have the most phenolic content as well as the best antioxidant properties. Microbial fermentation of DS flower also showed an increase in total phenolic content and DPPH scavenging activity of the fermented flower medium after the fermentation process. A recommendation for future research is perhaps to further identify the bioactive compound from the 100% methanolic crude extract of DS flower.

## Figures and Tables

**Table 1:** The extraction yield, TPC, and antioxidant capacity of crude extract.

Crude extract	Water (DSF WCE)	Methanol (DSF MCE)	Ethanol (DSF ECE)
Yield extract (%)	45.8	18.7	6.9
TPC (mg GAE/g extract)	31.64 ± 0.17	40.33 ± 1.47^*∗∗∗*^	34.50 ± 0.73^*∗*^

The TPC results are presented as mean ± SD. Post hoc (Tukey) test shows significant difference in TPC between DSF WCE and DSF ECE at *p* < 0.05 (*∗*) and significant difference between DSF WCE and DSF MCE at *p* < 0.001 (*∗∗∗*).

**Table 2:** The extraction yield, TPC, and antioxidant capacities of partitioned extract.

Crude extract using methanol	100% methanol (C 100)			Acidified methanol (C A)		
Yield extract (%)	18.9			22.5		
TPC (mg GAE/g extract)	26.82 ± 5.02			27.43 ± 0.38		

Further partition method	EA 100	W 100	H 100	EA A	W A	H A

Yield extract (%)	1.2	13.4	1.2	1.5	15.3	1.3
TPC (mg GAE/g extract)	171.03 ± 0.63^*∗∗∗*^	21.30 ± 1.80	17.46 ± 0.62	166.41 ± 2.35^*∗∗∗*^	18.66 ± 1.10	22.74 ± 1.30

The TPC results are presented as mean ± SD. Post hoc (Tukey) test shows no significant difference between TPC of 100% methanol (C 100) and acidified methanol (C A) crude extracts but shows significant difference in TPC between ethyl acetate extract (EA 100; EA A) and water layer extract (W 100; W A) and hexane extract (H 100; H A) at *p* < 0.001 (*∗∗∗*).
